# Insight into the Molecular and Structural Changes in Red Pepper Induced by Direct and Indirect Ultrasonic Treatments

**DOI:** 10.3390/molecules30244668

**Published:** 2025-12-05

**Authors:** Katarzyna Rybak, Aleksandra Skarżyńska, Szymon Ossowski, Magdalena Dadan, Katarzyna Pobiega, Małgorzata Nowacka

**Affiliations:** 1 Department of Food Engineering and Process Management, Institute of Food Sciences, Warsaw University of Life Sciences—SGGW, Nowoursynowska 159c, 02-776 Warsaw, Poland; katarzyna_rybak@sggw.edu.pl (K.R.); aleksandra_skarzynska@sggw.edu.pl (A.S.); szymon_ossowski@sggw.edu.pl (S.O.); magdalena_dadan@sggw.edu.pl (M.D.); 2 Department of Food Biotechnology and Microbiology, Institute of Food Sciences, Warsaw University of Life Sciences—SGGW, Nowoursynowska 159c, 02-776 Warsaw, Poland; katarzyna_pobiega@sggw.edu.pl

**Keywords:** red pepper (*Capsicum annuum* L.), ultrasound treatment, direct sonication, indirect sonication, quality attributes, SEM imaging, phytochemicals, bioactive compounds, FTIR spectroscopy, microbial quality

## Abstract

This study provides a comprehensive assessment of the effect of direct (probe) and indirect (bath) ultrasound treatments on the physicochemical and structural properties of red bell pepper (*Capsicum annuum* L.) tissue. Ultrasound was applied under controlled conditions to induce structural modification without excessive thermal or mechanical damage. The treated samples were evaluated using chemical (polyphenols, flavonoids, carotenoids, vitamin C, sugars), microbiological (total viable count (TVC) and total yeast and mold count (TYM)), spectroscopic (FTIR, NMR), thermal (TGA), and microscopic (SEM, micro-CT) analyses. Both ultrasound modes affected the tissue, but their effects differed in intensity and character. Direct ultrasound caused stronger cavitation and mechanical stress, resulting in greater cell wall disruption, higher permeability, and enhanced release of bioactive compounds such as polyphenols, vitamin C and antioxidants from the tissue matrix to the surroundings. Indirect ultrasound acted more gently, preserving cellular integrity and sugar profile while moderately increasing antioxidant activity. Cluster and correlation analyses confirmed that ultrasound mode was the main factor differentiating the samples. Short-term direct sonication enhanced the release of antioxidant compounds, whereas prolonged exposure led to their degradation, resulting in an overall decline in antioxidant capacity, and indirect ultrasound better preserved texture and sugar composition. This demonstrates that ultrasound mode and duration can be tailored to balance tissue integrity and enhance bioactive compounds in plant-based materials.

## 1. Introduction

Red bell pepper (*Capsicum annuum* L.) is an annual or biennial plant, valued for its unique taste and color. Its fruits are rich in bioactive components, such as polyphenols (mainly capsaicinoids), carotenoids (mainly capsanthin and capsorubin), vitamins B (niacin, pyridoxine), A, C, E, K and minerals (Na, K, Ca, Mg, P) [1,2,3]. The antioxidant properties of red pepper are especially pronounced. For instance, extracts of red *Capsicum annuum* exhibited protective effect on lipid peroxidation in the brain and liver, particularly due to Fe(II) chelating ability, scavenging of OH and NO radicals, and protection against over-stimulation of NMDA receptor [4].

The fruits of red bell peppers contain around 65–80% water, thus having a short shelf life. Although processing significantly reduces the spoilage of peppers, it can excessively degrade bioactive components, thereby limiting their antioxidant activity and nutrient content [5,6]. Therefore, it is crucial to select the processing method and its parameters to minimize unfavorable changes in the quality of peppers. One of the promising pretreatment methods is ultrasound (US), which can enhance conventional processing, contributing to improved product quality [7].

Ultrasound (US) technology can be defined as a series of sound waves above 18 kHz with an upper limit around 100 MHz [7,8,9]. The low-frequency range of US (usually 20–40 kHz) with acoustic intensity >1 W/cm^2^ is called power ultrasound and is typically used for structure modification and enhancement of different unit operations due to cavitation, “sponge effect”, and microstreaming [9,10,11]. Sonication pre-treatment can be applied (i) directly (using horns/probe mode or alternatively contact sonication) or (ii) indirectly using, above all, an ultrasonic bath [9,12]. The effect of direct sonication is predominantly much more noticeable, e.g., the rise in temperature is higher on account of the higher power intensity of the probe system and the small volume of the sample. Therefore, the time of direct sonication (probe) is shorter than the time of the indirect (bath) system and often requires a cooling unit [12]. The scientific literature lacks a sufficient, in-depth analysis of the impact of ultrasound on the quality aspects of red bell peppers, especially one that comprehensively compares different methods of ultrasound application—direct (probe) and indirect (bath).

Ultrasound may affect the quality of the material in a different way. Acoustic cavitation results in the disruption of the cells and the creation of microscopic channels in the structure [8,10,11]. This contributes to the acceleration of various processes but also releases components that are bound with the matrix [5,8], and thus the measured content of bioactives can be higher than in untreated material. On the other hand, during ultrasound treatment, free radicals are generated that can degrade bioactive components, such as vitamins or antioxidants, if the process parameters are not adjusted properly [11,13]. For instance, Nian et al. [5] reported that after sonication of chili pepper, the capsorubin content decreased by around 7% compared to the control sample, whereas capsaicin and dihydrocapsaicin content increased by 29% and 31%, respectively. At the same time, the vitamin C was degraded by 19% and the total phenolic content increased by 19%. US-assisted vacuum drying of green pepper decreased the total color difference up to 20%, as well as increased the total phenolic content and vitamin content up to 8 and 25%, respectively.

While probe (direct) vs. bath (indirect) ultrasound effects have been compared for several plant matrices (e.g., apples, lemon, carrots, tomatoes, etc.), these works typically focus on a subset of outcomes (juice quality, extraction yield, or drying kinetics) [14,15,16,17,18]. To our knowledge, no study has simultaneously combined chemical (TPC, TFC, vitamin C), structural (SEM, µCT), spectroscopic (FTIR, NMR), thermal (TGA), and microbiological analyses to compare direct and indirect ultrasound applied to red bell pepper (*Capsicum annuum* L.). Therefore, this study uniquely integrates multiple analytical techniques to provide a comprehensive comparison of the two sonication modes.

Different sonication times were applied to identify processing conditions that induce controlled structural modification without excessive thermal or mechanical damage. The treated samples were characterized using a multi-analytical approach, including chemical assays (polyphenols, flavonoids, antioxidant activity, carotenoids, vitamin C, sugars), spectroscopic techniques (Fourier-transform infrared spectroscopy—FTIR, nuclear magnetic resonance—TD NMR), thermal analysis (thermogravimetric analysis—TGA), microscopic analysis (scanning electron microscopy—SEM, microtomography), and colorimetric, textural, and microbiological analyses (total viable count—TVC, total yeast and mold count—TYM). Because the physicochemical properties of fresh plant tissues may vary across cultivars, origins, and maturity stages, the present study focused on a single pepper cultivar to isolate the effects of ultrasound. Nevertheless, variability among different pepper types may influence their response to sonication, and this warrants further investigation.

## 2. Results and Discussion

### 2.1. Temperature, Conductivity, and Cell Disintergration Index (CDI) During Ultrasound Treatment of Red Bell Pepper

As shown in Table 1, both direct (probe) and indirect (bath) ultrasound treatments caused moderate changes in the physicochemical parameters of the treatment medium. Throughout all experiments, the temperature of the medium was maintained within 20–25 °C, ensuring comparable processing conditions between treatments. In the case of direct ultrasound, to avoid a noticeable increase in temperature due to the high energy density of the probe system, the medium was periodically cooled using ice water to stabilize the temperature within the desired range. The temperature increased with the extension of the US time, reaching a maximum increase of 4.5 °C and 3.2 °C in the cases of direct and indirect ultrasound, respectively.

Electrical conductivity values also increased progressively with longer ultrasound exposure in both systems, reflecting enhanced ion diffusion from the pepper tissue into the surrounding medium, more pronounced for probe mode and almost unchanged for bath mode. Similar observations were made for the indirect sonication of apple and carrot up to 30 min [8]. A significant increase in conductivity was reported by the authors in the case of the apple when the treatment lasted more than 30 min. The calculated Cell Disintegration Index (CDI) also showed a gradual increase, confirming the occurrence of mild structural disruption of cellular membranes. More pronounced changes for direct ultrasound resulted from higher acoustic intensity and stronger cavitation effects compared to the indirect (bath) treatment. Jambrak et al. [19] similarly reported a higher increase in temperature in the case of direct sonication. However, they noted significantly higher electrical conductivity after bath treatment than probe one, regardless of the material (button mushrooms, Brussels sprouts, and cauliflower), due to placing samples directly into the cleaning bath. The authors explained that the tissue was not disrupted extensively after indirect ultrasound, and the conductivity rise after probe ultrasound was more likely to be acoustic cavitation.

The observed trends indicate that the selected ultrasound conditions induced controlled and reproducible modification of red bell pepper tissue, without excessive thermal or mechanical damage.

### 2.2. Physical Quality Attributes (Color and Texture)

Color is an important physical quality attribute. On the basis of color parameters (L*, a*, b*), the total color difference was calculated in comparison to the intact sample (control) and is presented in Table 2.

The color difference (ΔE) values indicated that ultrasound processing had a significant effect on the surface color of red bell pepper samples compared to fresh ones. Both the ultrasound mode (direct or indirect) and the exposure time influenced the extent of color changes observed on the top and bottom surfaces of the samples. In samples subjected to direct sonication, a progressive increase in ΔE was observed with longer treatment times, particularly on the bottom surface. The ΔE values for the skin ranged from 1.8 to 3.9, while those for the internal tissue varied between 5.5 and 17.8. Direct sonication induced substantial pigment alteration, likely due to intensified cavitation and localized heating effects. Such phenomena can disrupt cellular integrity and promote degradation of carotenoid pigments, including capsanthin and capsorubin, which are responsible for the characteristic red hue of red bell pepper [20]. The effect of indirect sonication was more heterogeneous. For the top surface, ΔE values ranged from 1.1 to 8.3, while for the bottom surface, they ranged from 9.5 to 17.8. The highest ΔE for the upper surface was observed after 5 min of treatment (ΔE = 8.3), whereas the lowest value was found for the 30 min treatment (ΔE = 1.1). This trend suggests that prolonged indirect sonication may mitigate color degradation, possibly by reducing the intensity of cavitation effects. Indirect sonication caused milder color changes; however, locally, particularly on the bottom surface of sample i_30 (ΔE =17.8), pronounced ΔE values were observed, likely due to uneven energy distribution within the ultrasonic bath or secondary oxidative processes occurring during extended exposure [21]. These findings suggest that the energy transfer mechanisms and cavitation dynamics differ between direct and indirect sonication modes, resulting in distinct effects on pigment stability and surface color.

The measured texture parameter, hardness (N), of the red bell pepper samples revealed that ultrasound treatment under the tested conditions exerted only minor effects on the mechanical firmness of the fruit tissue, compared with the fresh sample (Table 2). The fresh control exhibited a hardness of 52.3 N. In the direct ultrasound series, hardness values ranged from 48.5 N to approximately 52.7 N, indicating that only at the longest direct sonication time (5 min) was a slight decrease in firmness observed. In the indirect ultrasound series, the hardness ranged from 50.5 N to 55.8 N, with the highest value recorded at the 30 min indirect sonication, which was significantly higher than the values obtained from other treatments. The unexpected increase in hardness after 30 min of indirect sonication may be due to water redistribution and partial collapse of intercellular voids, which increases structural density. Prolonged indirect ultrasound may lead to mild structural re-arrangements, perhaps densification or collapse of intercellular spaces, which can increase resistance to deformation. Another possibility is sample-to-sample variation or inherent heterogeneity in the pepper tissues [22]. The limited variation in hardness suggests that, under the applied ultrasound parameters, the structural integrity of the pepper tissue was largely preserved. Although microstructural deformation and pore formation were clearly visible under SEM, these changes did not directly translate into a macroscopic loss of firmness, suggesting a compensatory structural rearrangement at the tissue level. This outcome is consistent with findings in recent studies, which emphasize that ultrasound pretreatments can improve firmness retention in fruits and vegetables when applied suitably [9]. In the present study, the slight decrease in hardness after 5 min of direct sonication may reflect the cumulative mechanical disruption of cell walls by cavitation, microjets, and shear forces near the ultrasound source. These micro-structural changes may reduce the resistance to deformation, albeit only modestly in our case. On the other hand, the slight increase in hardness observed for the 30 min indirect treatment (i_30) is not obvious. This is consistent with reports that low-power, indirect sonication mainly induces the “sponge effect” and limited degassing of intercellular spaces, improving sample compactness without extensive cell wall collapse. For leafy and soft vegetables subjected to power ultrasound through the surrounding medium, firmness losses are smaller than after contact/probe sonication, because the acoustic field is less focused and peak pressures are lower [23]. The results show that the way ultrasound modifies tissue hardness is determined mainly by the amount of acoustic energy reaching the sample and by the manner in which this energy is transmitted. High-intensity sonication with a probe, when applied for sufficiently long periods, produces a clear drop in firmness, which can be attributed to disruption of cell walls and a reduction in turgor caused by cavitation and local fluid movement. In contrast, ultrasound applied in a bath generates a less concentrated field and therefore acts more gently on the structure, which makes this variant suitable when maintaining the original texture is important. Such a distinction between intense contact sonication and milder immersion treatments has also been reported for other fruit and vegetable matrices processed with ultrasound and is reflected in the corresponding changes (or lack of changes) in hardness observed after subsequent operations [20,24].

### 2.3. Microbiological Quality

Ultrasound treatment noticeably reduced the natural microbiota of fresh pepper samples compared with the untreated control. The total viable count (TVC) of fresh red bell peppers was 4.57 ± 0.05 log CFU/g, while yeasts and molds (TYM) reached 3.51 ± 0.02 log CFU/g (Table 2). After direct sonication, a gradual decrease in microbial load was observed with increasing exposure time. TVC values dropped from 3.25 ± 0.26 log CFU/g after 0.5 min to 1.53 ± 0.17 log CFU/g after 5 min of treatment, corresponding to an approximate 3 log reduction. Yeasts and molds were below detectable levels after 3 min of direct exposure. Indirect ultrasound treatments produced even greater microbial reductions. TVC decreased from 2.19 ± 0.13 log CFU/g after 5 min to 1.00 ± 0.01 log CFU/g after 30 min. Similarly, TYM counts fell to nondetectable levels after 15 min of indirect sonication. These results demonstrate that ultrasound treatment, even in the absence of chemical sanitizers, can effectively inactivate the native microflora of pepper surfaces.

The observed microbial reductions can be attributed to acoustic cavitation effects. The rapid formation and collapse of cavitation bubbles generate localized micro-jets, shear stresses, and transient high-temperature/pressure zones that damage microbial cell walls, disrupt membranes, and enhance mass transfer. In the current study, the greater efficiency of indirect sonication may reflect a more uniform energy distribution within the treatment medium, allowing for longer exposure times without compromising product integrity. By contrast, shorter, more intense direct treatments produced similar but less homogeneous microbial reductions, likely due to energy dissipation near the probe surface [25]. Furthermore, despite indirect sonication having lower intensity, the prolonged exposure time in the ultrasonic bath (up to 30 min) provided microbial reductions comparable to or greater than those achieved by probe sonication, suggesting that total exposure time, rather than acoustic power density, governs microbial inactivation efficiency.

The extent of microbial reduction observed in this study (3–4 log CFU/g for TVC and >3 log CFU/g for TYM) is comparable to or superior to results reported in other ultrasound-based decontamination studies on fresh produce. Zhou et al. [26] demonstrated that ultrasound combined with sanitizers achieved reductions of 1.4–4.0 log in *E. coli* and *Salmonella* on grape tomatoes. Similarly, Alenyorege et al. [27] reviewed that ultrasound treatments alone often yield moderate reductions, but can achieve enhanced efficacy when combined with mild sanitizing agents or heat. Ultrasound treatments have previously been shown to inactivate bacteria and fungi on various vegetables such as lettuce, spinach, and tomatoes, though their efficacy is strongly dependent on sonication parameters (frequency, amplitude, medium, and time). From a technological perspective, the achieved microbial reductions are sufficient to meaningfully extend the microbiological shelf life of fresh peppers while maintaining acceptable texture and color quality. The data also suggest that indirect ultrasound treatment provides an optimal balance between decontamination efficacy and product preservation. These findings support the potential of ultrasound as a non-thermal, residue-free disinfection method for minimally processed vegetables [9].

Non-thermal methods for decontaminating peppers, such as ozonation, UV-C radiation, and pulsed electric field (PEF), differ in both the mechanism of microbial inactivation and the impact on product quality. For fresh and minimally processed peppers, the most thoroughly studied application of ozone—both in gaseous form and in ozonated water—has achieved reductions in total microbial counts of 2.0–3.5 log CFU/g with short exposure times, while maintaining the color, texture, and ascorbic acid content of freshly cut red peppers [28,29]. Despite its germicidal potential, UV-C caused only minimal immediate microbial reduction in fresh-cut pepper, and yeast and mold levels later matched the control. Although bacterial counts were temporarily lower in treated samples, final microbial loads (after 7 days of storage) were similar. Notably, reduced decay in UV-C–treated peppers suggests an effect on host susceptibility rather than strong direct decontamination [30]. A reduction in the number of microorganisms above 3 log cycles after the application of ultrasound indicates the possibility of placing ultrasound among the effective non-thermal sanitation methods for fresh produce.

### 2.4. Bioactive Compounds and Antioxidant Activity of Red Bell Pepper Treated with Direct and Indirect Ultrasound

#### 2.4.1. Bioactive Compounds (Total Polyphenols Content (TPC), Total Flavonoid Content (TFC), Total Carotenoid Content (TCC), Vitamin C Content) of Red Bell Pepper Treated with Direct and Indirect Ultrasound

**Figure 1:**
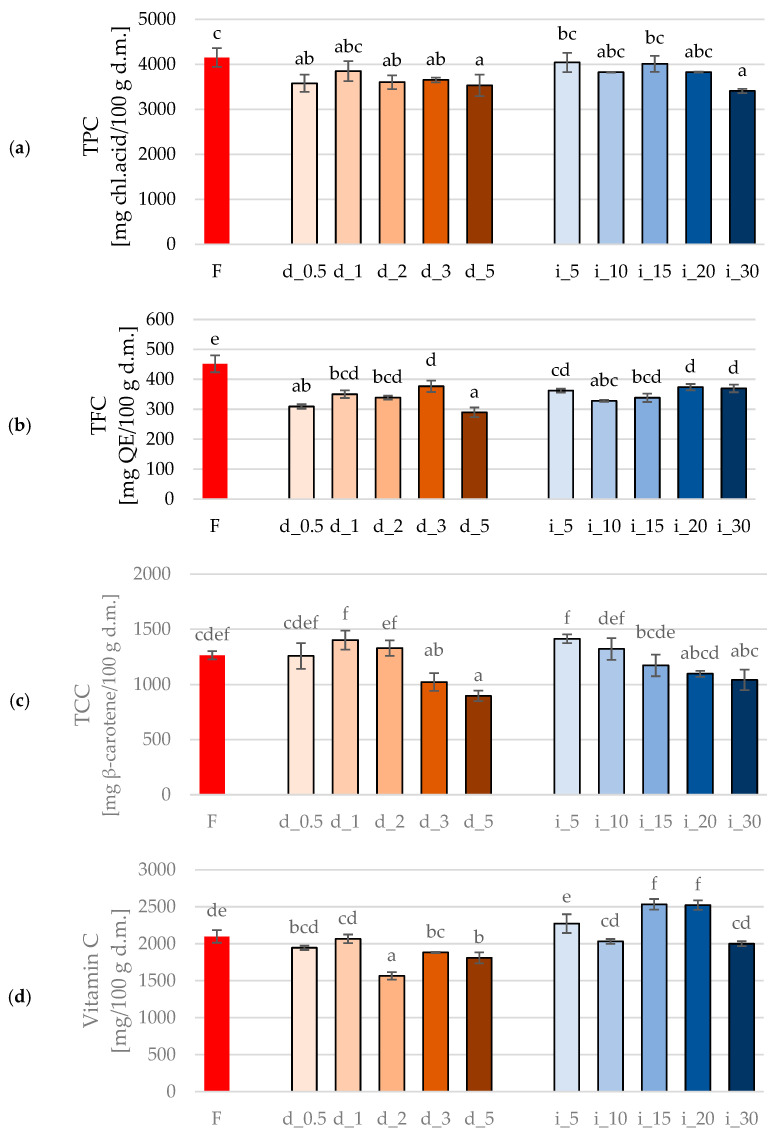
Effect of direct and indirect ultrasound treatment on bioactive compounds. (**a**) TPC; (**b**) TFC; (**c**) TCC; (**d**) Vitamin C in red bell pepper tissue. a–f—different letters above the columns indicate different homogeneous groups (α = 0.05).

Total Polyphenol Content (TPC) (Figure 1a) in fresh red bell pepper (F) reached the highest statistically significant value. Both direct and indirect ultrasonic treatments affected TPC, but the direction of change depended on sonication time and the type of ultrasound delivery.

In the case of the direct sonication system, a short exposure time (0.5–3 min; samples d_0.5–d_3) caused only a slight and statistically insignificant decrease in TPC compared to the control. This suggests that brief probe sonication induced limited structural disruption of the cellular matrix without significant degradation of polyphenolic compounds. The small observed fluctuations may be related to local cavitation effects, which increase cell wall permeability and facilitate the release of bound compounds. However, after 5 min of direct sonication (d_5), a significant reduction in TPC was observed, indicating that prolonged exposure to high-intensity acoustic energy caused degradation of phenolic constituents. This decrease can be attributed to the cumulative effects of strong cavitation, microstreaming, and localized heating near the probe, which enhance oxidation and thermal decomposition of the polyphenols [9,31,32,33].

Using an indirect ultrasonic system resulted in a statistically comparable level of TPC in samples treated for 5–20 min (i_5–i_20) to the fresh sample. This indicates that moderate acoustic energy transfer through the surrounding liquid medium did not significantly alter the TPC of the pepper tissue. However, for the longest treatment time (i_30), a noticeable and statistically significant decrease was observed, showing that excessive exposure time in the ultrasonic bath may also lead to the degradation of the studied polyphenolic compounds. Although the acoustic intensity of the bath system is lower, prolonged exposure increases cumulative cavitation and promotes a gradual temperature rise in the medium, which collectively facilitates oxidation reactions and breakdown of polyphenolic structures [34,35,36,37].

In the study by Tomšik et al. [38] extending the ultrasound treatment time for wild garlic increased the amount of extracted polyphenols. Similarly, in the study by Nian et al. [5], ultrasound treatment of chili peppers resulted in an increase in TPC by more than 19% compared to the control sample. On the other hand, Lučić et al. [20] demonstrated that extending the ultrasonic pretreatment time from 1 to 3 min for peppers subjected to drying caused a significant increase in TPC, whereas further extending the sonication duration to 5 min led to a subsequent decrease in TPC.

Figure 1b presents the TFC, and in fresh red pepper (F), TPC reached the highest value among all analyzed samples, indicating that every ultrasonic treatment, regardless of the system and treatment duration, led to a statistically significant reduction in TFC compared to the untreated material. In the series of samples treated with the ultrasonic probe, a clear range of TFC values can be observed.

The lowest flavonoid content was found in d_5, nearly 40% lower than in the fresh sample. Shorter sonication times (d_0.5, d_1, d_2) caused moderate but statistically significant decreases in TFC compared to the control. Sample d_3 showed the statistically highest flavonoid content among all direct variants, although it still remained clearly below the level of sample F. This can be interpreted as a narrow optimum of conditions in which intense cavitation improves the release of flavonoids from tissues before oxidative–thermal degradation begins to dominate. This phenomenon is consistent with the observations of other authors, who have shown that short-term ultrasound exposure increases the efficiency of phenolic and flavonoid compound extraction by loosening cell walls and facilitating diffusion, while prolonged sonication leads to the degradation of thermolabile bioactive components due to temperature rise and the generation of free radicals in cavitation zones [39,40].

In the series of samples from the ultrasonic bath, TFC values are more similar to each other, usually in the range of 320–380 mg QE/100 g d.m. For i_5 and i_20–i_30, relatively high TFC levels were obtained for sonicated samples, while i_10 and i_15 were slightly lower. The differences between bath variants are therefore smaller than in the probe system, suggesting that the ultrasonic bath delivers energy in a gentler and more uniform manner, so there is no sharp drop as in d_5. Such equalization of treatment effects has also been observed in other studies comparing bath and probe systems; probes generate higher power density and stronger cavitation, whereas baths more often lead to moderate changes in TPC and TFC [41,42].

Compared with the TPC profile in the same samples, flavonoids react somewhat more sharply to excessive sonication conditions: during longer probe sonication (d_5), the decrease in TFC is very pronounced, while TPC in some variants remains at a level close to the control. This is consistent with literature reports indicating that flavonoids, as a specific group of polyphenols, are particularly sensitive to elevated temperatures and prolonged ultrasound exposure, which leads to the degradation of aromatic rings and the loss of antioxidant activity [39,43,44].

As shown by Jedlińska et al. [45], an appropriate combination of ultrasound time and power (30 min, 160–200 W) favors the preservation of bioactive compounds in apples, while too short or excessive exposure leads to their degradation. A similar relationship was noted by Wang et al. [46] for *Codonopsis pilosula*, the flavonoid content increased up to 30 min of sonication and then decreased, confirming the existence of an energetic optimum of the process. Wang et al. [47] also subjected kiwi to ultrasound treatment (0–16 min at 400 W). Among other parameters, they examined flavonoid content and demonstrated that flavonoids in ultrasound-treated samples (16 min) increased by more than 105% compared to the control group.

Figure 1c presents the total carotenoid content (TCC) in red bell peppers, both untreated and treated with ultrasound (US). As can be seen, in most of the analyzed samples, no significant differences in TCC were detected between US-treated samples (regardless of the process method—direct or indirect) and the reference material (fresh red bell pepper). A significant reduction in total carotenoid content, compared to the unprocessed fresh sample, was observed only for samples d_3 and d_5. As can be seen in Table 1, these samples exhibited the highest values of the cell disintegration index (CDI), indicating the highest disruption of their tissue structure among all the samples tested. In general, conducting US treatment using a sonotrode, e.g., directly, is associated with more intense cavitation than in the indirect method, in which the effect of this phenomenon is weakened by a water bath (higher energy losses) [42]. This stronger mechanical impact of directly supplied ultrasound could indicate disruption of the cell membrane continuity, which could presumably result in increased leakage of some substances into the sonication medium [48,49,50].

The results of the vitamin C content analysis, shown in Figure 1d, indicate varying retention of this chemical compound depending on the process parameters used. Mildly processed red bell peppers to which ultrasound (US) was applied directly (d_0.5 and d_1) were characterized by the same vitamin C content as the reference material (fresh bell pepper). However, with increasing the intensity of US treatment (samples d_2, d_3, and d_5), lower vitamin C retention was observed. As mentioned above, these samples demonstrated the highest destruction of the tissue structure after US application (Table 1), which could have intensified the leaching of various substances from cellular structures along with cell sap into the medium [48,49,50]. Among all red bell peppers indirectly treated with US, two of the five analyzed samples differed in vitamin C content from fresh material (i_15 and i_20) and contained significantly more of it. In general, the indirect ultrasound treatment (bath) is considered to be gentler in material processing than the direct method (probe). This mild US treatment lasting 15 and 20 min may have been the optimal ultrasound operating range for sufficient tissue relaxation to facilitate the extraction of bioactive compounds in the subsequent operation, while reducing the risk of their leakage into the medium during US treatment [42]. It was observed that red bell peppers indirectly treated with ultrasound (bath) had higher vitamin C content than directly treated samples (probe). This may be related to the intensity of acoustic cavitation and subsequently to the amount of reactive oxygen species (ROS) created. Direct contact of the probe with the treated material is associated with the occurrence of intense cavitation, causing intense bubble collapse, local temperature increases, and generating large amounts of ROS, which degrade vitamin C through oxidation. This effect is mitigated by using an ultrasonic bath with a medium that, by suppressing acoustic energy, reduces the intensity of the cavitation phenomenon, thus minimizing the amount of ROS produced (lower risk/smaller scale of vitamin C oxidation) [7,51,52].

The differences in the stability of bioactive compounds between direct and indirect sonication are mainly due to different cavitation conditions and the scale of generation of highly reactive oxygen species (ROS). The implosion of bubbles in the sonotrode zone (at temperatures of several thousand Kelvin and pressures of hundreds of atmospheres) leads to the formation of high-temperature spots in the material exposed to US, where radicals are formed, responsible for the oxidative degradation of polyphenols and ascorbic acid [53,54,55,56]. In direct mode, the local power density is high, which intensifies both ROS production and structural damage to tissues. In the indirect system, the acoustic field is more diffuse, which limits the intensity of cavitation and reduces the exposure of bioactive compounds to oxidative stress [53,57]. This mechanistic model corresponds to the results obtained, where direct sonication led to faster degradation of TPC, TFC, and vitamin C than indirect sonication.

The retention profiles of bioactive compounds obtained in our study are not fully consistent with the results reported for other vegetables subjected to ultrasound treatment, which confirms that the effects of sonication are highly variable and depend not only on the raw material but also on the applied energy dose, exposure time, and configuration of the processing system. For carrot juices, it has been demonstrated that ultrasonic bath treatment (20–60 min) led to a statistically significant increase in TCC and vitamin C content [58]. In the study by Cuéllar-Villarreal et al. [59], whole carrots were exposed to ultrasound using a sonotrode (400 W, 24 kHz, amplitude 100 mm, 5 min). In direct response to ultrasound, carrots showed more than a 21% higher carotenoid level compared with fresh samples, which, according to the authors, suggests that ultrasound enhanced the extractability of these compounds. Ultrasonic treatment of carrots resulted in a decrease in TPC by more than 62%. However, after 3 days of storage, the TPC in the sonicated samples increased by nearly 130%, while in the non-sonicated samples by less than 19%. The authors concluded that the increase in TPC observed in ultrasound-treated samples may be related to a stress response induced by the ultrasound treatment.

Similar results were observed for romaine lettuce subjected to ultrasound (25 kHz) [60]. After 2.5 days of storage, a 35% (60 s) and 27% (120 s) increase in polyphenol content was recorded compared with control samples. In the study by Esua et al. [61], tomatoes treated with ultrasound combined with UV-C radiation for 20 min exhibited a 50% higher polyphenol content than the control (fresh tomatoes). The authors attributed this effect to the release of bound phenolics and the activation of phenylalanine ammonia-lyase (PAL), an enzyme responsible for the synthesis of specific phenolic compounds such as flavonoids, chlorogenic acids, coumarins, and phenylpropanoids. Lu et al. [62] demonstrated that ultrasound treatment of commercial tomatoes (25 kHz, acoustic power density of 26 W/L) enhanced the accumulation of secondary metabolites, including total polyphenols, lycopene, carotenoids, and ascorbic acid, during storage for up to 48 h.

#### 2.4.2. Antioxidant Activity of Red Bell Pepper Treated with Direct and Indirect Ultrasound

**Figure 2:**
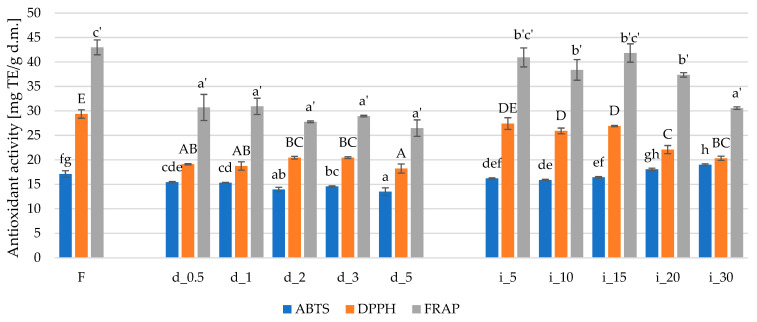
Effect of direct and indirect ultrasound treatment on antioxidant activity of red bell pepper tissue. a–h, A–E, a′–c′—Different letters above the columns indicate different homogeneous groups for ABTS, DPPH, and FRAP, respectively (α = 0.05).

In the studied system, the antioxidant activity of red bell pepper, as assessed by ABTS, DPPH, and FRAP assays, depended significantly on the type and duration of sonication (Figure 2). Direct ultrasound (d_0.5–d_5) resulted in a gradual decrease in all three indicators—most notably for DPPH and FRAP, which reached their lowest values after 5 min of sonication (d_5). In contrast, during indirect sonication (i_5–i_30), antioxidant activity was better preserved: for DPPH and FRAP, samples i_5–i_15 did not differ statistically or differed only slightly from the fresh sample, whereas ABTS clearly increased with treatment duration, reaching its maximum in the i_30 variant. This pattern indicates that the gentler effect of the ultrasonic bath promotes the preservation or even enhancement of antioxidant capacity, while the high power density in the probe system with prolonged exposure may lead to the degradation of active compounds [63,64].

Correlation analysis confirmed a strong relationship between bioactive composition and antioxidant capacity. Total polyphenol content correlated (Table S1) strongly and positively with DPPH (r = 0.774, *p* = 0.009) and FRAP (r = 0.842, *p* = 0.002), as well as with vitamin C (r = 0.700, *p* = 0.024). Meanwhile, ABTS showed a significant, though weaker, correlation mainly with vitamin C (r = 0.633, *p* = 0.050), with no significant relationships with TPC or TFC. This indicates that, in the studied samples, the results of DPPH and FRAP tests were primarily driven by the set of phenolic compounds, whereas in the case of ABTS, the greater contribution came from the fraction of low-molecular-weight hydrophilic antioxidants, mainly ascorbic acid.

Differences between the tests can be further explained by their reaction mechanisms. Both DPPH and FRAP are based mainly on single-electron transfer (SET), reacting preferentially with electron donors of phenolic structure, whereas ABTS combines SET and hydrogen atom transfer (HAT) mechanisms and is more sensitive to the presence of small, water-soluble molecules [65,66,67]. Consequently, the decrease in TPC observed during prolonged direct sonication translated into a clear reduction in DPPH and FRAP values, while ABTS—more closely linked to vitamin C—remained relatively stable or even increased in the indirect variants, in which the highest vitamin C content was recorded (i_15, i_20).

The use of ultrasound as a pretreatment method for plant materials significantly affects their antioxidant activity, and the effect depends on process parameters and the type of raw material. Calderón-Martínez et al. [68] demonstrated that short-term sonication of Purslane (Portulaca oleracea) pulp increased antioxidant activity measured by ABTS and DPPH methods and reduced vitamin C loss, indicating that moderate doses of acoustic energy can stabilize bioactive compounds. Similar results were obtained by Assad et al. [69], who found that longer ultrasound treatment of purslane, especially when combined with freeze-drying, allowed the retention of up to 95% of total antioxidant activity and high phenolic content. In contrast, Ren et al. [70] reported an opposite effect for onions, where prolonged sonication prior to hot-air drying caused polyphenol degradation and a decrease in antioxidant activity.

### 2.5. Sugars and Molecular Profile

#### 2.5.1. Sugars (Sucrose, Glucose, Fructose) Content in Red Bell Pepper Treated with Direct and Indirect Ultrasound

**Figure 3:**
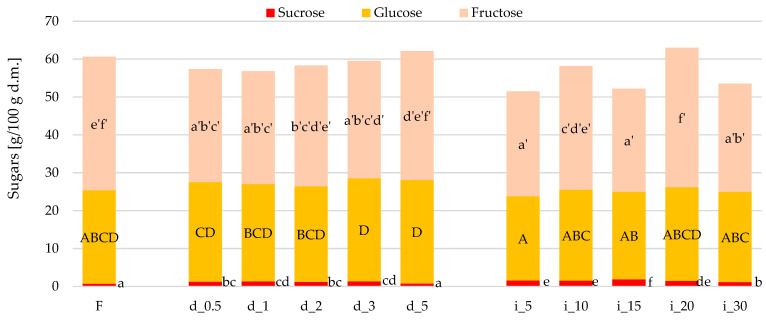
Effect of direct and indirect ultrasound treatment on sugars content in red bell pepper tissue. a–f, A–D, a′–f′—Different letters above the columns indicate different homogeneous groups for sucrose, glucose, and fructose, respectively (α = 0.05).

The content of the disaccharide sucrose and its two constituent monosaccharides—glucose and fructose [71]—in untreated and ultrasound-treated red bell peppers is shown in Figure 3. The unprocessed fresh sample (F) contained 0.7, 24.6, and 35.3 g/100 g d.m. sucrose, glucose, and fructose, respectively. Regardless of the method of ultrasound treatment (direct or indirect) or the duration of this process, US-treated red bell peppers exhibited more sucrose (up to 2.5 times more—sample i_15), a comparable amount of glucose, and, in most cases analyzed, a lower fructose content. Ultrasound treatment likely contributed to increased disaccharide (sucrose) extractability by appropriately opening the structures within the tissue, while simultaneously preventing leakage of this substance into the medium [72,73]. Comparing two ultrasound treatment methods, samples treated with direct US had, on average, lower sucrose content but higher glucose and fructose content than samples treated with indirect US. In general, direct ultrasound is a more intensive treatment than indirect ultrasound [42], which means that the scale of physicochemical changes in the treated material may also be higher. Certain reports indicate that ultrasound can affect the release and activity of invertase, an enzyme responsible for the conversion of sucrose to glucose and fructose [71,74,75], which is present in fresh red bell pepper tissue [76]. For example, Vargas et al. [77] studied the effect of direct US (probe) on the release and activity of invertase from *Aspergillus niger* cultivated in various substrates. The authors concluded that US caused cell disruption and invertase release. Increased activity of this enzyme was also observed. Soares et al. [78] demonstrated that indirect US (bath) negatively affects the structure of invertase and reduces its activity. These observations may potentially explain the tendencies discovered in this study. Increased invertase activity may enhance the conversion of sucrose to glucose and fructose.

#### 2.5.2. FTIR Spectra of Red Bell Pepper Treated with Direct and Indirect Ultrasound

**Figure 4:**
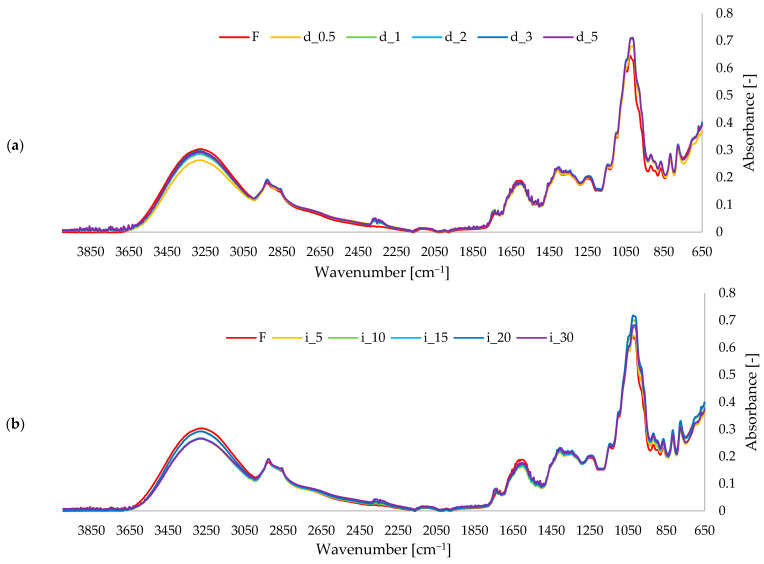
Effect of (**a**) direct and (**b**) indirect ultrasound treatment on FTIR spectra of red bell pepper tissue.

FTIR spectra recorded for the red pepper samples (Figure 4) showed a band arrangement typical of dehydrated plant material. A broad absorption in the 3600–3000 cm^−1^ region was attributed to O–H stretching vibrations of structural water, cell-wall polysaccharides, and phenolic constituents. Two bands at approximately 2920 and 2850 cm^−1^ corresponded to asymmetric and symmetric C–H stretching of aliphatic groups. The signal observed at around 1740 cm^−1^ was assigned to C=O stretching of esterified components and lipid/carotenoid fractions naturally present in pepper pericarp, whereas the band near 1630 cm^−1^ was related to absorbed water and to C=C or C=O vibrations of low-molecular plant metabolites. In the fingerprint region (1200–900 cm^−1^), the spectra were dominated by intense C–O–C and C–O stretching bands originating from cell-wall polysaccharides, which is consistent with FTIR characterizations of plant matrices [79,80]. Comparison of the control spectrum with spectra of ultrasound-treated peppers did not reveal qualitative differences, indicating that the applied sonication conditions did not modify the principal functional groups of the matrix. Only minor intensity changes were noted at 1740 cm^−1^ and within 1150–1030 cm^−1^, e.g., in spectral areas sensitive to the organization and exposure of carbohydrate and esterified structures. Such low-amplitude variations can be interpreted as the effect of a partial microstructural rearrangement (greater accessibility of existing components) rather than as evidence of chemical transformation. The FTIR results, therefore, confirm that ultrasound, in the present experimental setup, influenced mainly the structural arrangement of pepper tissue, while its molecular profile remained essentially unchanged [79,80,81].

#### 2.5.3. NMR of Red Bell Pepper Treated with Direct and Indirect Ultrasound

**Figure 5:**
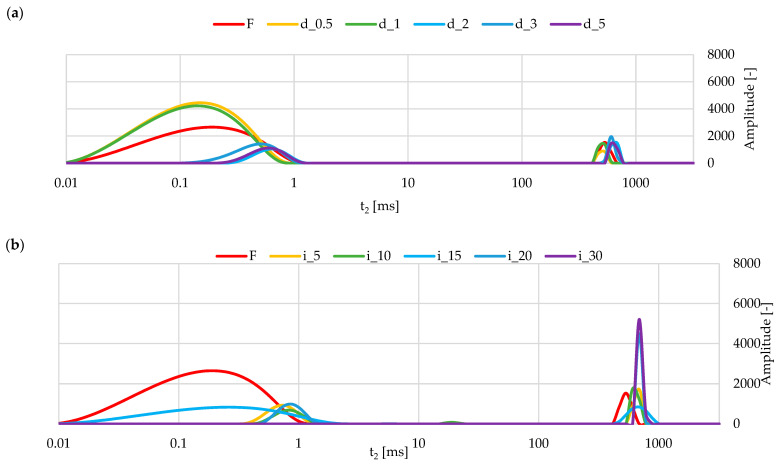
Effect of (**a**) direct and (**b**) indirect ultrasound treatment on T_2_ spectra (examples) of red bell pepper tissue.

**Figure 6:**
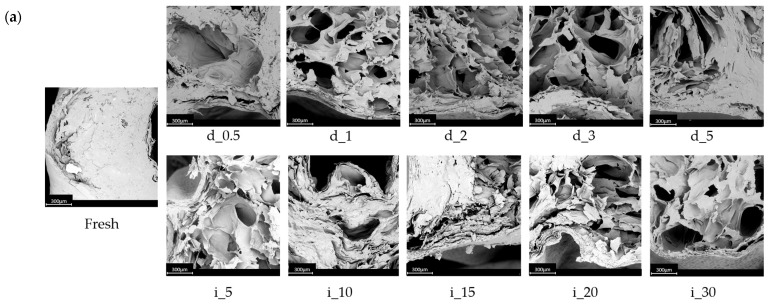

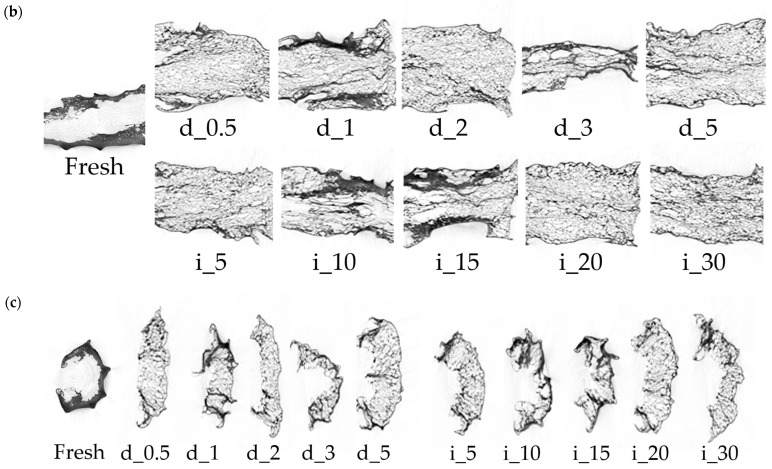
Effect of direct and indirect ultrasound treatment on structural changes of red bell pepper tissue, (**a**) photographs from SEM (magnification 200×); (**b**) cross-section micro-CT visualization; (**c**) micro-CT visualization of whole sample (raw images inverted in black and white colors to facilitate observation).

The TD-NMR analysis (Figure 5a,b) showed that ultrasound treatment modified the distribution of water populations in pepper tissue. The control sample (Figure 5a) exhibited a typical relaxation profile of plant parenchyma with three components corresponding to tightly bound water, intracellular water, and a fraction of more mobile water, as previously reported for fruit tissues subjected to mild processing [82]. After sonication with the probe, the relaxation curves (Figure 5b) shifted toward shorter T_2_ values, and the main peak decreased in amplitude, indicating an increase in the proportion of water relaxing at newly exposed solid surfaces and thus a higher degree of structural disruption. A comparable shortening of T_2_ in response to high-energy ultrasound has been described for structured food systems, attributed to cavitation-related damage and enlargement of the interfacial area [8,83]. In turn, samples treated in the ultrasonic bath showed only moderate peak broadening and slight displacement with respect to the control (Figure 5b), which can be interpreted as a partial opening of the tissue without complete loss of water within the tissue. Hence, the TD-NMR results confirm that the effect of ultrasound on pepper tissue was dependent on the sonication mode and the intensity of acoustic energy transferred to the sample. These results were confirmed by structural changes (Figure 6). The shortening of T_2_ values, observed particularly for direct ultrasound, corresponds to the increased internal surface area and fragmentation revealed by SEM and micro-CT. Water molecules interact more strongly with newly exposed solid surfaces, which accelerates the relaxation process. In indirect ultrasound, milder structural loosening results in moderate T_2_ changes, consistent with smaller pore enlargement seen by SEM and micro-CT.

### 2.6. Thermal and Structural Characteristics

#### 2.6.1. TGA of Red Bell Pepper Treated with Direct and Indirect Ultrasound

**Figure 7:**
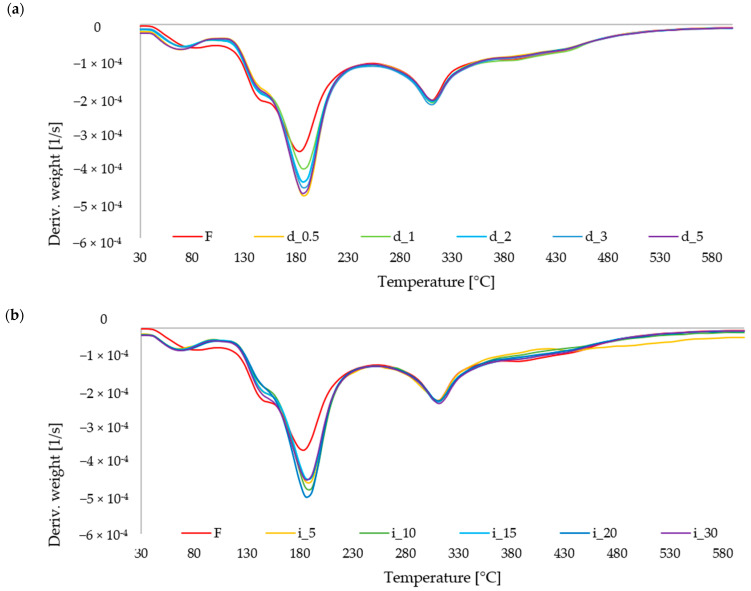
Effect of (**a**) direct and (**b**) indirect ultrasound treatment on TGA of red bell pepper tissue.

Thermogravimetric analysis (Figure 7) was used to evaluate whether ultrasound affected the thermal stability of the pepper tissue. All samples showed a three-step mass-loss pattern typical of plant materials heated under nitrogen: an initial region up to about 120 °C, corresponding to removal of physically bound water; a major decomposition stage between approx. 200 and 350 °C, related to the breakdown of polysaccharide fractions (hemicellulose- and pectin-rich domains); and a slower tail above 350 °C, attributed to the degradation of more ordered cellulose structures and to residual organic matter. A comparable course of TG/DTG curves has been reported for dried peach and other plant matrices analyzed under N_2_ in the range 30–600 °C [84,85,86]. In the present study, ultrasound-treated peppers exhibited slightly higher mass loss in the first stage and, for probe-sonicated variants, a minor shift in the main DTG peak toward lower temperatures (Figure 7b). This indicates that sonication increased the accessibility of moisture and partially opened the cell-wall network, which reduced the energy required to initiate thermal degradation. Similar peak shifts were observed when structural pretreatments or high-energy processes were applied prior to drying of peach slices and were interpreted as a consequence of microstructural loosening and increased specific surface area [87]. At the same time, the final residues at 600 °C remained comparable, indicating that ultrasound did not significantly alter the mineral fraction or the total amount of thermally stable components. Overall, the TGA/DTG profiles confirm that the effect of ultrasound on pepper was structural rather than compositional, consistent with earlier descriptions of thermally induced mass loss in plant tissues subjected to mechanical or physical pretreatments [88,89].

#### 2.6.2. Structural Changes of Red Bell Pepper Treated with Direct and Indirect Ultrasound Evaluated on the Basis of SEM and Micro-CT

Scanning electron microscopy (SEM) and X-ray microcomputed tomography (micro-CT) analyses were performed to visualize the microstructural alterations in red bell pepper tissue induced by ultrasound treatment. The fresh sample exhibited a compact and well-organized cellular structure with small and uniformly distributed intercellular spaces, typical of a hydrated and intact red bell pepper (Figure 6).

The micro-CT and SEM results clearly showed that direct (probe) ultrasound treatment produced a more porous internal structure of red bell pepper tissue compared to the indirect (bath) system.

After ultrasound treatment, noticeable structural changes were observed, especially in samples subjected to direct sonication (d_0.5–d_5), which exhibited progressively more pronounced microstructural disintegration, which was confirmed by higher CDI (see Table 1). SEM micrographs revealed the formation of irregular pores, ruptured cell walls, and collapsed intercellular spaces, particularly after longer sonication times (≥ 3 min) (Figure 6a). These effects are associated with acoustic cavitation phenomena, including microstreaming and localized shock waves, which generate strong shear stresses that disrupt plant tissue. Similar observations have been reported for ultrasound-treated fruits and vegetables such as carrots, apples, and tomatoes, where the collapse of parenchymal cells and the development of microchannels facilitated mass transfer processes [63,90]. The micro-CT reconstructions further supported these findings, showing a gradual increase in porosity and structural deformation in the direct ultrasound samples (Figure 6b,c). Cross-sectional views indicated the presence of large, interconnected pores corresponding to gas cavities formed during cavitation (Figure 6b). This suggests a significant alteration of the internal structure, which may affect diffusion and water mobility, and this is consistent with the LF-NMR results indicating changes in T_2_ relaxation components (see Figure 5).

In contrast, the tissue treated with indirect ultrasound (i_5–i_30) maintained a more compact and continuous structure, with smaller and evenly distributed pores. SEM images showed partial loosening of the cell network and minor detachment of the cuticle, while most cellular compartments remained intact. The micro-CT images revealed a more homogeneous structure with smaller, evenly distributed pores, confirming that the energy transfer in the ultrasonic bath was less intense and more uniform compared to the probe system. These observations are consistent with previous findings. Studies comparing the two sonication modes have demonstrated that probe ultrasound delivers higher localized energy density, generating stronger cavitation and mechanical stress, whereas bath sonication promotes a more uniform but gentler treatment, primarily causing limited cell wall loosening and soft tissue expansion rather than rupture. Similar trends were reported for plant and biopolymer matrices, where bath sonication produced fewer disruptions and smoother surface morphology compared to direct probe sonication [91,92].

The combined SEM and micro-CT analyses demonstrate that the system mode of ultrasound treatment and the time of the treatment alter the red bell pepper tissue. Direct ultrasound leads to severe mechanical stress, cell disruption, and pore formation, whereas indirect ultrasound causes a smaller impact, resulting in gradual relaxation of the structure and partial rearrangement of the tissue matrix. Such controlled modification of plant microstructure can be advantageous in food processing applications, as it enhances mass transfer, improves extractability of bioactive compounds, and facilitates drying or infusion processes [63].

### 2.7. Overall Impact of Ultrasound Type on Red Bell Pepper Tissue

**Figure 8:**
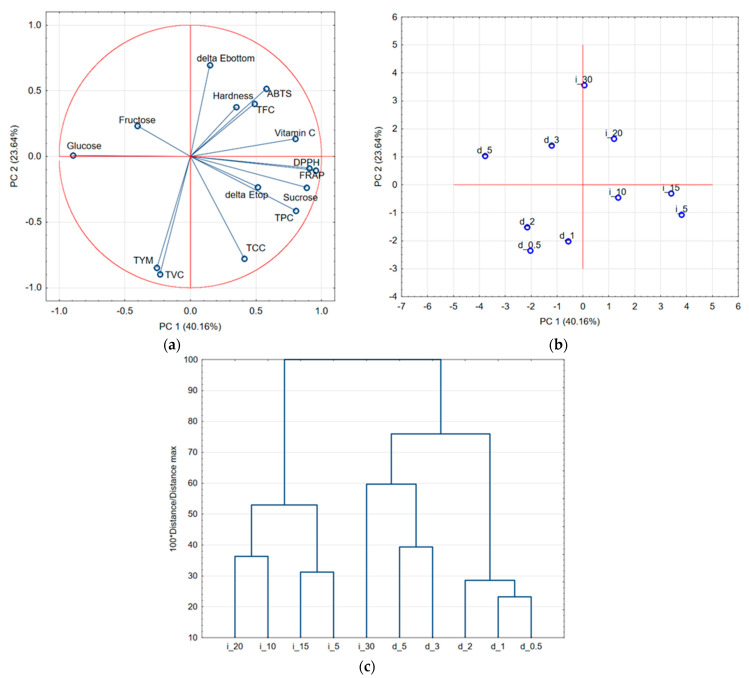
Principal Component Analysis (PCA), (**a**) loading plot showing relationships among physicochemical, biochemical, and microbiological parameters; (**b**) score plot illustrating the distribution of samples according to ultrasound mode and treatment duration; (**c**) hierarchical cluster analysis (Ward’s method, Euclidean distance) of red bell pepper samples treated with direct and indirect ultrasound.

Ultrasound treatment noticeably influenced the physicochemical and biochemical properties of red bell pepper tissue, with distinct effects observed between direct and indirect sonication modes (Figure 8).

Cluster analysis revealed four major clusters, which separated the samples according to ultrasound mode and exposure time. Indirect ultrasound variants (i_5, i_10, i_15, i_20) formed a compact group with moderate biochemical response, while direct ultrasound variants (d_0.5, d_1, d_2) clustered separately, indicating stronger structural disruption and enhanced release of bioactive compounds. The d_3 and d_5 samples grouped together, reflecting stronger sonomechanical disruption accompanied by enhanced phenolic release and antioxidant activity, whereas indirect i_30 created a separate group, which was quite close to the d_3 and d_5 samples. This behavior of indirect treatment for 30 min (i_30) may be related to cumulative cavitation and prolonged acoustic treatment, which enhance water penetration into the tissue and promote gradual swelling and loosening of cell walls. Similar long-term cavitation phenomena and hydration-driven structural relaxation have been previously reported for potato [93]. Principal Component Analysis provided a clear visualization of the impact of ultrasound mode and intensity on the biochemical and structural attributes of red bell pepper tissue. The first two principal components, PC1 (40.16%) and PC2 (23.64%), explained 63.8% of the total variance. The loading plot (Figure 8a) revealed strong positive correlations among antioxidant-related parameters (ABTS, DPPH, FRAP, TFC, TPC, and vitamin C) and color attributes (ΔEtop, ΔEbottom, and TCC), while microbial counts (TVC, TYM) were negatively associated with these variables. The score plot (Figure 8b) clearly separated the samples according to the mode of sonication. Indirectly sonicated samples (i_5, i_10, i_15, i_20, i_30) were positioned on the positive side of PC1, closely related to antioxidant and color variables, indicating enhanced antioxidant retention and carotenoid stability. In contrast, directly sonicated samples (d_0.5–d_5) clustered on the negative side of PC1, correlating with higher microbial loads and lower antioxidant activity. Direct sonication induced stronger biochemical responses, though it is not necessarily beneficial for product quality. In contrast, indirect sonication produced milder yet more desirable changes, preserving pigment stability and structural integrity. Thus, PCA demonstrated that indirect sonication exerted a more favorable effect on the antioxidant, color, and microbiological quality of the samples compared to direct sonication. It should be emphasized that all ultrasound treatments in the present study were conducted at a laboratory scale. Numerous studies indicate that industrial ultrasound systems differ substantially from laboratory devices in terms of reactor geometry, acoustic energy distribution, cavitation intensity, and thermal profiles, which may alter mass transfer, structural modifications, and bioactive compound stability [90,94]. Therefore, further research using pilot- or industrial-scale flow-through reactors is required to confirm whether the effects observed here can be reproduced under commercial processing conditions.

## 3. Materials and Methods

### 3.1. Materials

The plant material used in this study was red bell pepper (*Capsicum annuum* L.), cultivar ‘California Wonder’, purchased at a local wholesale market (Bronisze, Poland). To minimize natural variability, all red bell peppers used in this study originated from the same commercial batch, representing a single cultivar and uniform maturity stage. This approach was chosen to ensure that all observed differences could be attributed to ultrasound treatment rather than biological variation. The peppers were stored under refrigerated conditions (85–95% relative humidity, 4–5 °C) until analysis. Only undamaged fruits with uniform external appearance, firmness, and size were selected for the experiment. Prior to analysis, peppers were washed in cold water, blotted to remove surface moisture, deseeded, and the pericarp was cut into 2 × 4 cm sections. The thickness of the obtained slices was maintained at approximately 5 mm. For each ultrasound treatment and for the control, 150 g of pepper tissue were used. The tissue pieces originated from approximately six individual red bell peppers of similar size and color.

### 3.2. Ultrasonic Treatment

**Figure 9:**
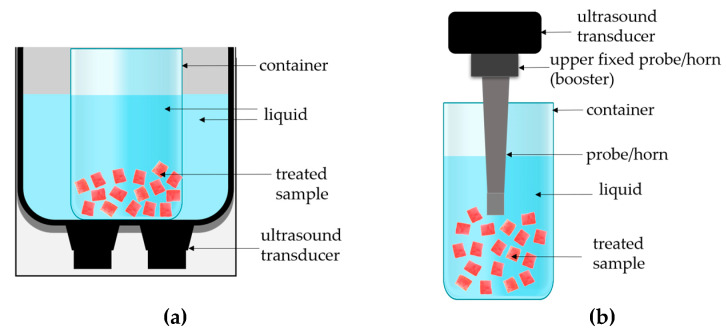
Schematic representation of (**a**) indirect (bath) and (**b**) direct (probe) ultrasound treatments applied to red bell pepper tissue.

Two ultrasound-assisted methods were applied to modify the pepper tissue prior to subsequent analyses: indirect sonication (ultrasonic bath, 21 kHz, 300 W, MKD Ultrasonics, Stary Konik, Poland) and direct sonication (probe ultrasound, 24 kHz, 400 W, UP400S, Hielscher Ultrasonics GmbH, Teltow, Germany). In these systems, acoustic energy was transmitted to the sample in different ways (Figure 9).

#### 3.2.1. Indirect Sonication (Ultrasonic Bath System)

The indirect mode of ultrasound treatment was carried out using an ultrasonic bath (Figure 9a). In this system, transducers located at the bottom of the tank convert electrical energy into high-intensity sound waves, which propagate through the liquid medium (water) surrounding the sample. The ultrasound energy is transmitted indirectly to the plant tissue through the liquid, without physical contact between the transducer and the material. During the treatment, the red bell pepper samples were fully immersed in water at a sample-to-liquid ratio of approximately 1:4 to ensure uniform transmission of the ultrasound waves. For indirect sonication, samples were placed at the bottom of a glass beaker (01L) filled with a constant volume of water (600 mL), which acted as the transmission medium. The beaker was placed centrally in an ultrasonic bath with transducers mounted at the bottom of the bath. Reproducibility was ensured by maintaining constant water volume (600 mL), sample mass (150 g), water depth (to the line given by the bath producer), and identical positioning of the beaker for all treatments. The processing time varied between 5 and 30 min (samples i_5–i_30). Based on preliminary trials, we observed that direct probe sonication caused a rapid increase in temperature. Therefore, throughout all experiments, the sample beaker was placed inside a larger beaker filled with an ice–water mixture to stabilize the temperature. The temperature in the sample was monitored with a digital thermometer during treatment, and additional ice was added when necessary. Although continuous temperature recording was not performed, the temperature was controlled manually and maintained below 25 °C for all treatments. Short temporary rises above this value were avoided by replenishing the ice–water medium. This approach ensured consistent and reproducible thermal conditions during direct sonication. After sonication, samples were drained and used for further analyses.

#### 3.2.2. Direct Sonication (Ultrasonic Probe System)

Direct ultrasound treatment was conducted using a high-intensity ultrasonic probe (horn-type sonotrode) operating at 24 kHz and 400 W (UP400S, Hielscher, Teltow, Germany) (Figure 9b) with an amplitude of 100%. The probe (12 mm diameter) was immersed 15 mm below the liquid surface. Each sample consisted of 600 mL of medium and 150 g of pepper tissue. Probe position and geometry were kept constant to ensure reproducibility. In this configuration, the sonotrode tip is submerged directly in the sample medium, providing direct mechanical coupling between the transducer and the red bell pepper tissue. The horn converts the electrical signal into longitudinal mechanical vibrations, generating a highly localized field of intense acoustic cavitation in the liquid surrounding the tissue.

Compared with the indirect system, the probe delivers higher acoustic power density, resulting in stronger cavitation, microjetting, and localized heating. Consequently, the treatment time was shorter (0.5–5 min; samples d_0.5–d_5) and a cold bath was used to prevent excessive temperature rise and structural degradation. The amplitude and immersion depth of the probe were optimized to achieve homogeneous energy distribution while avoiding overheating.

Preliminary experiments were conducted to evaluate the influence of ultrasound duration and mode on temperature, electrical conductivity, and the calculated Cell Disintegration Index (CDI) for red bell pepper. The temperature of the medium was monitored using a thermometer. After each ultrasound exposure time, the electrical conductivity of the surrounding medium was measured using a CPC-505 conductometer (Elmetron, Zabrze, Poland). The obtained conductivity values were used to calculate the Cell Disintegration Index (CDI), which expresses the relative degree of cellular disruption caused by ultrasound treatment. These measurements provided complementary information on the extent of ultrasound-induced tissue disintegration, enabling quantitative comparison between the effects of direct and indirect sonication modes. Total cellular destruction was achieved through three freeze–thaw cycles (−18 °C for 24 h, followed by thawing at 25 °C for 4 h), where conductivity plateaued after the third cycle, confirming complete membrane rupture. The CDI was determined based on the ratio between the conductivity of the medium after treatment and that of the fully disrupted tissue (after threefold freezing (−18 °C) and thawing), according to the following equation [95]:

CDI = (C_t_ − C_0_)/(C_max_ − C_0_), (1)

where C_t_ is the conductivity measured after a given treatment time, C_0_ is the conductivity of the medium before treatment, and C_max_ represents the conductivity obtained after total cellular destruction.

**Figure 10:**
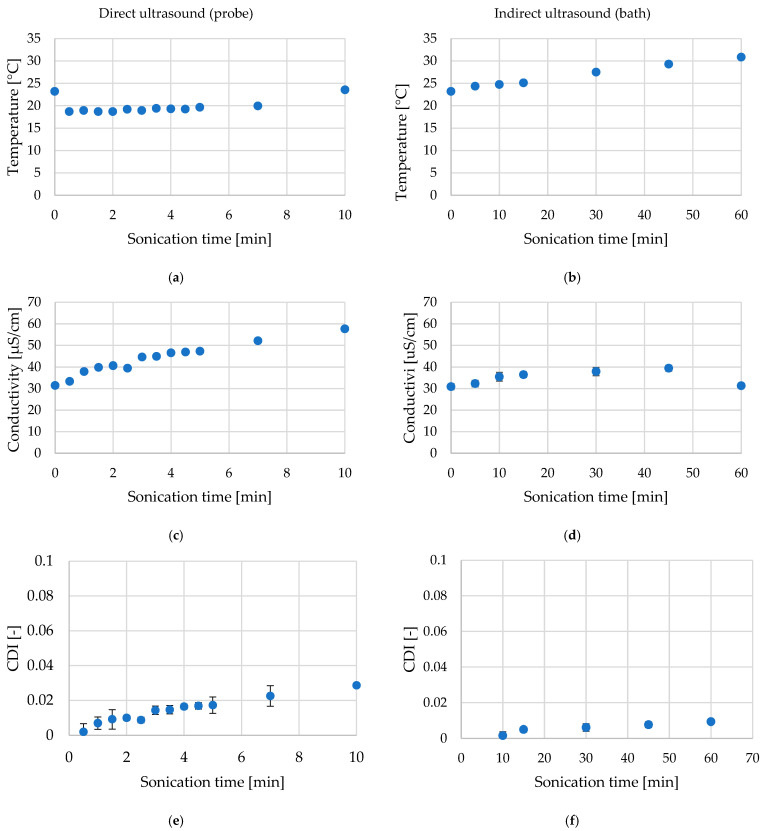
The effect of ultrasound treatment for direct sonication on (**a**) temperature, (**c**) conductivity, (**e**) CDI, and for indirect sonication on (**b**) temperature, (**d**) conductivity, (**f**) CDI.

The data were monitored across a wider range of processing times, 5–60 min for the indirect (bath) treatment, and 0.5–10 min for the direct (probe) treatment (Figure 10).

Based on preliminary data, ultrasound conditions that did not exhibit substantial temperature differences were selected for further analysis. This ensured that subsequent physicochemical, structural, and microbiological investigations were performed on samples, minimizing the risk of thermal or mechanical overprocessing. The parameters used to select the final ultrasound conditions are summarized in Table 3.

Consequently, the following variants were chosen for detailed examination:

- Direct ultrasound treatment (probe system): d_0.5, d_1, d_2, d_3, and d_5 (0.5–5 min),
- Indirect ultrasound treatment (bath system): i_5, i_10, i_15, i_20, and i_30 (5–30 min),
- The fresh (untreated) sample (F) was used as a control in all comparative analyses.

### 3.3. Research Methods

#### 3.3.1. Color

Color measurements of red bell pepper were carried out using the reflectance method in the CIE Lab* color space with a CR-5 colorimeter (Konica Minolta, Tokyo, Japan). The instrument was operated with a 2° standard observer, D65 illuminant, and an 8 mm measurement aperture. Prior to measurements, the colorimeter was calibrated against manufacturer-supplied white and black reference tiles with fixed L*, a*, and b* values. For each treatment, color was determined on both the external surface (exocarp) and the internal surface (cut side) of the pepper. Six independent readings were taken for each surface, repositioning the probe each time to account for sample heterogeneity. The results are expressed as L* (lightness), a* (green–red axis), and b* (blue–yellow axis). The overall color difference (ΔE) between the treated samples and the fresh pepper was calculated according to [96]:

∆*E* = [(∆L*)^2^ + (∆a*)^2^ + (∆b*)^2^]^0.5^ (2)

where L* is lightness, a* is the chromatic coordinate on the red (+)/green (−) axis, b* is the chromatic coordinate on the yellow (+)/blue (−) axis, and ΔL*, Δa*, Δb* denote the differences in these coordinates between the sonicated and the control (fresh) pepper samples.

Macroscopic images of the external surface and internal side of the pepper were recorded using a digital camera Nikon D7000 (Nikon, Tokyo, Japan) equipped with a 105 mm lens. The camera was positioned 50 cm from the sample. Illumination was provided by four fluorescent daylight lamps (6500 K) arranged at a 45° angle to the sample. All photographs were taken in a shadow-free (light-diffusing) chamber to ensure uniform lighting and to minimize reflections.

#### 3.3.2. Texture

Texture profile analysis (TPA) of red bell pepper tissue was performed using a TA.XT2i texture analyser (Stable Micro Systems, Surrey, UK) [97]. From each sample, cylindrical specimens (10 mm diameter) were cut with a cork borer to obtain pieces of uniform diameter and height, and each specimen was placed centrally under the compression probe. The analyser operated in compression (distance) mode with the following settings: pre-test speed, 1 mm/s, test speed, 5 mm/s, post-test speed, 1 mm/s, penetration depth, 10 mm, and automatic force triggering at 50 g. Each specimen was subjected to two consecutive compression cycles, separated by a 5 s interval. Hardness (the first maximum force) was selected as the primary TPA descriptor and was calculated as the maximum force attained during the first compression cycle. For every processing variant, ten measurements were carried out.

#### 3.3.3. Water State in Samples

TD-NMR was used to characterize the water distribution in fresh and ultrasound-treated red peppers. Measurements were performed on a 20 MHz benchtop spectrometer (minispec mq20, Bruker, Rheinstetten, Germany). Cylindrical tissue pieces (≈10 mm diameter, 10–12 mm height) were cut from the central part of the fruit, placed in 10 mm NMR tubes and analyzed at 22 ± 1 °C. Transverse relaxation (T_2_) was measured with the CPMG pulse sequence (echo time 0.3 ms, 6000 echoes, recycle delay 10 s, 8 scans) [98]. About 100 relaxation points were collected over 1–3000 ms to capture both short and long components. The decay curves were converted to T_2_ distributions by multi-exponential (inverse Laplace) analysis and shifts in T_2_ values and peak areas were used to evaluate the effect of ultrasound mode and treatment time on tissue structure. All samples were analyzed in triplicate.

#### 3.3.4. Total Polyphenols Content (TPC)

For the determination of total phenolics, total flavonoids and antioxidant activity, the plant material was first comminuted in an analytical mill (IKA A11 basic, IKA-Werke GmbH, Staufen, Germany). Extraction was carried out with 80% (*v*/*v*) ethanol: weighed samples were mixed with the solvent and shaken for 12 h at 16 °C on a laboratory shaker (Multi Reax, Heidolph Instruments, Schwabach, Germany). The extracts were then clarified by centrifugation at 4350 rpm for 4 min (MegaStar 600, VWR, Leuven, Belgium), and the supernatants were used for spectrophotometric assays.

Total phenolic content was determined by the Folin–Ciocalteu method. An aliquot of the extract (10 µL) was placed in a 96-well microplate and diluted with distilled water, followed by the addition of 40 µL of fivefold-diluted Folin–Ciocalteu reagent. After 3 min, 250 µL of 7% (*w*/*v*) Na_2_CO_3_ was added. The reaction mixtures were incubated for 60 min at room temperature in the dark, and absorbance was read at 750 nm using a Multiskan Sky microplate reader (Thermo Electron, Waltham, MA, USA) [99]. Calibration was performed with chlorogenic acid (0–100 µg mL^−1^, R > 0.999), and the results are expressed as mg chlorogenic acid equivalents per 100 g dry matter. All measurements were carried out in triplicate.

#### 3.3.5. Total Flavonoid Content (TFC)

Total flavonoid content was determined by the aluminum chloride colorimetric method [100]. An aliquot of the sample extract (20 µL) was placed in a 96-well microplate and mixed with 80 µL of distilled water and 10 µL of 5% (*w*/*v*) NaNO_2_ solution. After 5 min, 10 µL of 10% (*w*/*v*) AlCl_3_ was added, and the mixture was allowed to react for a further 6 min. Subsequently, 40 µL of 1 mol L^−1^ NaOH was added, the contents were thoroughly mixed, and the volume was adjusted with distilled water to give a measurable absorbance. After 20 min of incubation at room temperature, absorbance was read at 510 nm using distilled water as a reference. Quantification was carried out using a quercetin calibration curve (0–500 µg mL^−1^, R > 0.998), and the results are expressed as mg quercetin equivalents per 100 g dry matter. All samples were analyzed in triplicate.

#### 3.3.6. Total Carotenoid Content (TCC)

Total carotenoid content (TCC) was determined spectrophotometrically according to a procedure adapted from PN-EN 12136:2000 [101]. Ground pepper (1.5 g) was clarified with 20 mL of water and 1 mL each of Carrez I and II, centrifuged (5 min, 2000× *g*), and the residue was extracted three times with 20 mL of acetone. The combined acetone extracts were partitioned with 40 mL of petroleum ether, phase separation was facilitated by adding 10 mL of water, and the ether layer was dried over 1.5 g of anhydrous Na_2_SO_4_. The clear extract was made up to 100 mL with petroleum ether and its absorbance was read at 450 nm (Spectronic 200, Thermo Fisher Scientific Inc.). TCC was calculated as β-carotene equivalents using the extinction coefficient 2592 for petroleum ether. All determinations were performed in triplicate.

#### 3.3.7. Vitamin C Content

Vitamin C was determined by UPLC using an ACQUITY UPLC H-Class system (Waters, Milford, MA, USA) equipped with a PDA detector, following the UHPLC-PDA conditions described by Spínola et al. [102]. To minimize degradation, extraction was carried out immediately after sampling and under reduced light. Portions of fresh pepper tissue (0.5 g) were weighed into chilled tubes and homogenized with 10 mL of a cold extraction medium containing 3% (*w*/*v*) metaphosphoric acid and 8% (*v*/*v*) acetic acid. The homogenates were vortexed for 10 min and centrifuged at 6000 rpm for 5 min at 4 °C. To enable determination of total vitamin C (ascorbic acid + dehydroascorbic acid), the supernatant was mixed 1:1 with a dithiothreitol (DTT) solution (1 g L^−1^) and incubated for 60 min at 4 °C. After reduction, the extracts were filtered through 0.22 µm PSF GHP syringe filters (Pall, Ann Arbor, MI, USA). Chromatographic separation was performed on an ACQUITY UPLC HSS T3 column (2.1 × 100 mm, 1.8 µm; Waters, Drinagh, Ireland) with a 5 µL injection volume. The column oven was kept at 25 °C, the autosampler at 4 °C, and the mobile phase was delivered at 0.25 mL min^−1^. Detection was set at 245 nm. Quantification was carried out using an external calibration curve prepared from L-ascorbic acid standards in the concentration range 0.005–0.10 mg mL^−1^ (R > 0.999). All determinations were performed in triplicate.

#### 3.3.8. Antioxidant Activity (DPPH, ABTS, FRAP)

Antioxidant capacity was evaluated using DPPH• and ABTS•^+^ radical scavenging assays in a microplate format [75]. Stock radical solutions were prepared 24 h before analysis. The DPPH• solution was obtained by dissolving 25 mg of 2,2-diphenyl-1-picrylhydrazyl (Sigma-Aldrich, St. Louis, MO, USA) in methanol. The ABTS•^+^ solution was prepared by dissolving 38.4 mg of ABTS (2,2′-azino-bis(3-ethylbenzothiazoline-6-sulfonic acid)) and 6.6 mg of potassium persulfate in 10 mL of distilled water and allowing the mixture to stand in the dark (room temperature, 16–24 h) to complete radical generation. Immediately prior to measurement, both radical solutions were diluted with 80% (*v*/*v*) ethanol to the working absorbance: 0.68–0.72 at 515 nm for DPPH• and at 734 nm for ABTS•^+^. The reactions were carried out in 96-well plates by adding 10 µL of the ethanolic sample extract to 250 µL of the appropriate radical solution. For the DPPH• assay, plates were incubated for 30 min in the dark and absorbance was read at 515 nm. For the ABTS•^+^ assay, absorbance was measured after 6 min at 734 nm. In both cases 80% ethanol was used as the reference (blank). Radical scavenging activity was calculated from the decrease in absorbance of the radical solution in the presence of the extract and expressed as Trolox equivalents (mg Trolox g^−1^ dry matter) on the basis of a Trolox calibration curve prepared in 80% ethanol.

Ferric reducing antioxidant power (FRAP) was measured following the procedure described by Xiao et al. [103], with freshly prepared reagents. The FRAP working solution was prepared by mixing 0.3 mol L^−1^ acetate buffer (pH 3.6), 10 mmol/L TPTZ in 40 mmol/L HCl, and 20 mmol/L FeCl_3_·6H_2_O in the ratio 10:1:1, and preheating the mixture to 37 °C. In a 96-well plate, 180 µL of the working solution was mixed with 5 µL of the sample extract, shaken, and incubated for 15 min at 37 °C in the dark. Absorbance was recorded at 593 nm using distilled water as a blank. Trolox standards were analyzed under the same conditions, and antioxidant capacity was expressed as µmol/Trolox g dry matter. All samples were analyzed in triplicate.

#### 3.3.9. Sugar Content

Total sugars were analyzed by HPLC fitted with a refractive index detector (Waters, Milford, MA, USA). Fresh material (1.0 g) was weighed into screw-cap tubes and extracted with 10 mL of Milli-Q water (18.2 MΩ·cm) at 80 °C for 4 h on a Multi Reax shaker set to 1200 rpm. The hot extracts were clarified by centrifugation at 4350 rpm for 2 min, and the resulting supernatants were passed through 0.22 µm Acrodisc PSF GHP syringe filters (Pall Life Sciences, New York, NY, USA). Carbohydrates were separated on a Sugar-Pak I cation-exchange column (6.5 × 300 mm, 10 µm; Waters, Milford, CT, USA) equipped with a Sugar-Pak Guard-Pak cartridge, maintained at 90 °C. Isocratic elution with deionized water was applied at 0.6 mL min^−1^, and 10 µL of sample were injected; the chromatographic run time was 20 min [104]. Sucrose, D-glucose, and D-fructose (Sigma-Aldrich, Steinheim, Germany) were used to build external calibration curves in the 0–5000 µg/mL range (R^2^ > 0.999). All samples were analyzed in triplicate.

#### 3.3.10. FTIR

Infrared spectra were acquired with a Cary 630 FTIR instrument (Agilent Technologies, Santa Clara, CA, USA) equipped with a single-bounce diamond ATR accessory. Dried samples were placed directly on the ATR crystal and gently pressed to ensure good contact. For the samples, spectra were recorded from 4000 to 650 cm^−1^ at 4 cm^−1^ spectral resolution, co-adding 64 scans to improve the signal-to-noise ratio [105]. Before measuring the next sample, a new background spectrum was collected and the crystal surface was cleaned with isopropanol. Spectra were collected and processed in MicroLab FTIR (Agilent), and every sample was analyzed in triplicate; the averaged spectrum was used for further evaluation.

#### 3.3.11. TGA

Thermal behavior of the samples was evaluated by thermogravimetric analysis using a TGA/DSC 3+ instrument (Mettler Toledo, Greifensee, Switzerland). About 6 mg of dried material was weighed into 70 µL alumina pans and heated from 30 to 600 °C at a constant rate of 5 °C min^−1^. Measurements were carried out under a nitrogen purge of 50 mL/min to provide an inert atmosphere. During the run, mass loss (TG) and the corresponding rate of mass loss (DTG) were registered and subsequently used to distinguish the individual stages of thermal decomposition and to determine their characteristic peak temperatures. TG/DTG curves were processed in STARe Evaluation Software (v. 16.10, Mettler Toledo, Parramatta, Australia). All determinations were performed in duplicate to confirm repeatability of the analysis.

#### 3.3.12. Microbial Analysis

The red bell pepper (10 g) was mixed with 90 mL 0.85% NaCl and then homogenized (Stomacher 400 Circulator, Seward Limited, Cambridge, UK) for 30 s. Total viable count (TVC) was enumerated on plate count agar (PCA) incubated at 30 °C for 72 h. Yeasts and molds (TYM) were counted on Dichloran Rose-Bengal Chloramphenicol (DRBC) agar after incubation at 25 °C for 120 h. The number of microorganisms was counted using ProtoCOL 3-device (Synbiosis, Frederick, MD, USA) and determined in log CFU/g [106]. All determinations were conducted in triplicate, the homogenization step ensured that TVC and TYM results represent total microbiota (surface and internal). All media were purchased from Biomaxima, Lublin, Poland.

#### 3.3.13. SEM and Microtomography

The red bell pepper microstructure was assessed by scanning electron microscopy (SEM) and X-ray microcomputed tomography (µCT) [45]. For SEM, dried samples were fixed on aluminum stubs with double-sided tape and sputter-coated with 5 nm gold (Leica EM ACE200, Leica, Vienna, Austria). Observations were carried out in a Phenom XL microscope (Thermo Fisher Scientific, Waltham, MA, USA) at 10 kV and ~10 Pa; for the samples, four cross-sectional photos were acquired at 200× magnification. For µCT, tissue pieces (~2 × 3 cm) were mounted on a 25 mm holder and scanned in a Skyscan 1272 system (Bruker, Kontich, Belgium) at 40 kV, 193 µA, 0.3° rotation steps over 180°, with 13.3 µm voxel size. Projection data were binarised in CTAn (Bruker) and reconstructed in NRecon (Bruker) to obtain central cross-sections and 3D views of the internal structure.

### 3.4. Statistical Analysis

All experimental data are expressed as mean values ± standard deviation and all statistical analyses were performed using Statistica 13.3 (TIBCO Software Inc., Palo Alto, CA, USA). The results were statistically evaluated using one-way analysis of variance (ANOVA) to determine significant differences among sonication treatments at a significance level of α = 0.05, followed by Tukey’s post hoc test to identify homogeneous groups.

Principal Component Analysis (PCA) was performed on standardized data. The following parameters were included in the PCA model: Total Polyphenol Content (TPC), Total Flavonoid Content (TFC), ABTS, DPPH, FRAP, Vitamin C, Sucrose, Glucose, Fructose, Total Carotenoid Content (TCC), color parameters (ΔE_TOP, ΔE_BOT), Hardness, Total Viable Count (TVC), and Total Yeast and Mold Count (TYM). Additionally, to assess similarities and differences between samples treated with direct and indirect ultrasound, Cluster Analysis (CA) was performed using Ward’s linkage method and Euclidean distance as a measure of dissimilarity. This allowed identification of natural groupings based on the combined physicochemical and biochemical variables. Pearson’s correlation coefficients (r) were calculated to evaluate relationships between selected chemical, antioxidant, and textural parameters. Correlations were considered statistically significant at *p* < 0.05.

## 4. Conclusions

The study demonstrated that both direct and indirect ultrasound treatments significantly altered the physicochemical, biochemical, and structural properties of red bell pepper tissue. However, the extent and character of these changes were strongly dependent on the ultrasound mode. Direct (probe) ultrasound induced more pronounced effects, resulting in higher cell disruption, greater permeability, and enhanced release of bioactive compounds such as polyphenols and vitamin C, accompanied by a reduction in firmness and partial degradation of thermolabile constituents. In contrast, indirect (bath) ultrasound provided a gentler treatment, effectively preserving the native microstructure and sugar composition while maintaining antioxidant potential.

Cluster and correlation analyses confirmed that the mode of ultrasound application was the main discriminating factor. The observed structural loosening, as verified by SEM and micro-CT imaging, supports the potential of ultrasound to alter tissue permeability, which can be used for subsequent processing based on mass and heat transfer. Ultrasound can be strategically applied to balance structural preservation and bioactive release in red pepper tissue. Direct sonication enables rapid structural modification and release of compounds but may accelerate degradation at longer times, while indirect sonication provides a gentler, more uniform treatment, preserving pigment stability, antioxidants, and microbial quality. These findings indicate that ultrasound parameters can be optimized to achieve controlled modification of plant tissue, allowing for obtaining functional attributes in plant-based foods while minimizing degradation of bioactive compounds. However, this study evaluated the effects of ultrasound using a single pepper cultivar and laboratory-scale equipment. As natural variability among cultivars or industrial-scale cavitation conditions may alter the response to ultrasound, future work should address these factors to establish the broader applicability of the findings.

## Figures and Tables

**Table 1:** Temperature, electrical conductivity, and CDI values of red bell pepper tissue after direct and indirect ultrasound treatment.

Type of Ultrasound	Sample Code	Time of Treatment [min]	Medium Temperature [°C]	Conductivity [µS/cm]	CDI [-]
Fresh	F	0	23.2 ± 0.28 ^ab^	30.9 ± 1.4 ^a^	- *
Direct ultrasound	d_0.5	0.5	18.7 ± 0.28 ^a^	33.4 ± 2.5 ^ab^	0.0020 ^a^
	d_1	1	19.0 ± 0.07 ^a^	37.9 ± 4.2 ^bc^	0.0070 ^a^
	d_2	2	18.7 ± 0.28 ^a^	40.6 ± 5.1 ^cd^	0.0099 ^ab^
	d_3	3	23.2 ± 0.28 ^ab^	44.6 ± 1.5 ^de^	0.0143 ^bc^
	d_5	5	23.2 ± 0.28 ^ab^	47.3 ± 1.7 ^e^	0.0173 ^c^
Indirect ultrasound	i_5	5	24.3 ± 0.07 ^ab^	32.4 ± 1.4 ^ab^	0.0015 ^a^
	i_10	10	24.8 ± 0.07 ^b^	35.5 ± 2.0 ^abc^	0.0050 ^a^
	i_15	15	25.1 ± 0.14 ^c^	36.5 ± 1.3 ^bc^	0.0061 ^a^
	i_20	20	-	- *	-
	i_30	30	27.5 ± 0.14 ^d^	37.9 ± 1.9 ^bc^	0.0077 ^ab^

* Data not available; ^a–e^—different letters in the same column indicate different homogeneous groups (α = 0.05).

**Table 2:** Color (ΔE—total color difference of top and bottom and real color from the photo of the top and bottom), texture (hardness), and microbial quality (TVC, TYM) of red bell pepper tissue after direct and indirect ultrasound treatment.

Type of Ultrasound	Sample Code	Color [-]				Texture	Microbial Quality	
		ΔE Top	ΔE Bottom	Color of Top	Color of Bottom	Hardness [N]	TVC [log CFU/g]	TYM [log CFU/g]
Fresh	F	- *	-	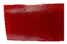	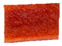	52.3 ± 4.5 ^ab^	4.57 ± 0.05	3.51 ± 0.02
Direct ultrasound	d_0.5	1.9 ± 0.9 ^abc^	5.5 ± 0.9 ^a^	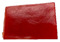	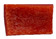	50.4 ± 4.1 ^ab^	3.25 ± 0.26	3.38 ± 0.04
	d_1	2.1 ± 1.0 ^abc^	8.4 ± 2.0 ^ab^	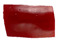	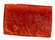	52.7 ± 2.7 ^ab^	3.13 ± 0.10	3.10 ± 0.02
	d_2	1.8 ± 0.9 ^ab^	11.8 ± 1.0 ^cde^	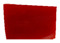	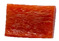	52.0 ± 3.0 ^ab^	3.10 ± 0.07	2.61 ± 0.05
	d_3	2.9 ± 1.5 ^abc^	8.8 ± 2.3 ^bc^	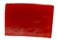	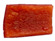	52.2 ± 4.6 ^ab^	2.35 ± 0.05	nd ^#^
	d_5	3.9 ± 1.1 ^bc^	13.0 ± 2.9 ^e^	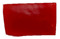	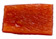	48.5 ± 4.5 ^a^	1.53 ± 0.17	nd
Indirect ultrasound	i_5	8.3 ± 1.1 ^d^	14.6 ± 0.4 ^ef^	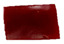	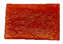	52.1 ± 4.7 ^ab^	2.19 ± 0.13	1.30 ± 0.25
	i_10	2.8 ± 1.6 ^abc^	11.4 ± 1.4 ^bcde^	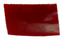	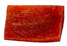	53.8 ± 2.5 ^ab^	2.15 ± 0.11	1.16 ± 0.22
	i_15	4.1 ± 1.4 ^c^	12.4 ± 2.3 ^de^	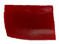	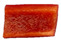	50.5 ± 4.8 ^ab^	2.05 ± 0.18	nd
	i_20	1.6 ± 0.6 ^a^	9.5 ± 1.6 ^bcd^	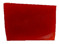	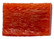	51.5 ± 4.6 ^ab^	1.36 ± 0.32	nd
	i_30	1.1 ± 0.9 ^a^	17.8 ± 0.8 ^f^	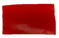	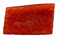	55.8 ± 3.2 ^b^	1.00 ± 0.01	nd

* No data due to the ΔE was calculated in comparison to the fresh sample, ^#^ nd—not detected, ^a–f^—different letters in the same column indicate different homogeneous groups (α = 0.05).

**Table 3:** Parameters for direct and indirect ultrasound treatments of red bell pepper tissue and their selection.

Type of Ultrasound	Tested Time Range [min]	Temperature Range During Experiments [°C]	CDI Range [-]	Reason for Selection
Direct ultrasound (probe)	0.5–10	16.0–23.6 (with cooling)	0.003–0.029	Higher times caused strong tissue damage (high CDI), which could bias chemical results, 0.5–5 min ensured structural modification without overheating.
Indirect ultrasound (bath)	5–60	23.2–30.1	0.0007–0.009	Long times (>30 min) resulted in gradual temperature increase and structural softening; 5–30 min ensured mild treatment.

## Data Availability

The original contributions presented in this study are included in the article/Supplementary Material. Further inquiries can be directed to the corresponding author.

## Supplementary Materials

The following supporting information can be downloaded at: https://www.mdpi.com/article/10.3390/molecules30244668/s1, Table S1. Pearson’s correlation coefficients between physicochemical, antioxidant, and structural parameters of red bell pepper tissue treated with direct and indirect ultrasound.
