# Supplementary material for: Insight into the Molecular and Structural Changes in Red Pepper Induced by Direct and Indirect Ultrasonic Treatments
Source: Molecules. 2025 Dec 5;30(24):4668. doi: 10.3390/molecules30244668 (PMC12735565; doi:10.3390/molecules30244668)
Supplement: Supplementary file 1 [file molecules-30-04668-s001.zip › molecules-4008719-supplementary.pdf]

molecules

MDPI

Supplementary Materials

Table S1. Pearson's correlation coefficients between physicochemical, antioxidant, and structural parameters of red bell pepper tissue treated with direct and indirect ultrasound

Parameter	TPC	TFC	ABTS	DPPH	FRAP	Vitamin C	Sucrose	Glucose	Fructose	TCC	ΔE top	ΔE bottom	Hardness	TVC	TYM
TPC	1.0000	.2236	.0864	.7743	.8427	.7007	.8300	-.5962	-.2231	.5521	.6231	-.1800	-.0894	.1460	.0040
	p= ---	p=.535	p=.812	p=.009	p=.002	p=.024	p=.003	p=.069	p=.535	p=.098	p=.054	p=.619	p=.806	p=.687	p=.991
TFC	.2236	1.0000	.5751	.2346	.2686	.3599	.4220	-.3591	-.1159	.0922	-.0223	.4637	.6304	-.2425	-.2841
	p=.535	p= ---	p=.082	p=.514	p=.453	p=.307	p=.224	p=.308	p=.750	p=.800	p=.951	p=.177	p=.051	p=.500	p=.426
ABTS	.0864	.5751	1.0000	.3076	.4876	.6328	.3740	-.6085	-.1260	-.0353	-.1715	.1508	.6584	-.5824	-.3381
	p=.812	p=.082	p= ---	p=.387	p=.153	p=.050	p=.287	p=.062	p=.729	p=.923	p=.636	p=.677	p=.038	p=.077	p=.339
DPPH	.7743	.2346	.3076	1.0000	.9238	.6060	.8216	-.8368	-.3275	.3855	.6223	.1787	.1680	-.1741	-.2726
	p=.009	p=.514	p=.387	p= ---	p=.000	p=.063	p=.004	p=.003	p=.356	p=.271	p=.055	p=.621	p=.643	p=.631	p=.446
FRAP	.8427	.2686	.4876	.9238	1.0000	.8376	.8810	-.8281	-.2501	.3911	.5049	-.0588	.1581	-.1960	-.2214
	p=.002	p=.453	p=.153	p=.000	p= ---	p=.002	p=.001	p=.003	p=.486	p=.264	p=.137	p=.872	p=.663	p=.587	p=.539
Vitamin C	.7007	.3599	.6328	.6060	.8376	1.0000	.7256	-.5846	-.0927	.0593	.2814	-.1112	.0681	-.3791	-.3781
	p=.024	p=.307	p=.050	p=.063	p=.002	p= ---	p=.018	p=.076	p=.799	p=.871	p=.431	p=.760	p=.852	p=.280	p=.281
Sucrose	.8300	.4220	.3740	.8216	.8810	.7256	1.0000	-.6892	-.3750	.4885	.3145	-.0675	.2096	.0989	-.0631
	p=.003	p=.224	p=.287	p=.004	p=.001	p=.018	p= ---	p=.027	p=.286	p=.152	p=.376	p=.853	p=.561	p=.786	p=.863
Glucose	-.5962	-.3591	-.6085	-.8368	-.8281	-.5846	-.6892	1.0000	.4515	-.4858	-.4411	-.1500	-.4347	.2481	.1245
	p=.069	p=.308	p=.062	p=.003	p=.003	p=.076	p=.027	p= ---	p=.190	p=.155	p=.202	p=.679	p=.209	p=.489	p=.732
Fructose	-.2231	-.1159	-.1260	-.3275	-.2501	-.0927	-.3750	.4515	1.0000	-.3638	-.3844	-.2412	-.2646	-.2396	-.1901
	p=.535	p=.750	p=.729	p=.356	p=.486	p=.799	p=.286	p=.190	p= ---	p=.301	p=.273	p=.502	p=.460	p=.505	p=.599
TCC	.5521	.0922	-.0353	.3855	.3911	.0593	.4885	-.4858	-.3638	1.0000	.2624	-.4281	.2105	.6586	.7345
	p=.098	p=.800	p=.923	p=.271	p=.264	p=.871	p=.152	p=.155	p=.301	p= ---	p=.464	p=.217	p=.559	p=.038	p=.016
ΔE top	.6231	-.0223	-.1715	.6223	.5049	.2814	.3145	-.4411	-.3844	.2624	1.0000	.2322	-.2400	-.0247	-.1300
	p=.054	p=.951	p=.636	p=.055	p=.137	p=.431	p=.376	p=.202	p=.273	p=.464	p= ---	p=.518	p=.504	p=.946	p=.720

ΔE bottom	-.1800	.4637	.1508	.1787	-.0588	-.1112	-.0675	-.1500	-.2412	-.4281	.2322	1.0000	.3964	-.5549	-.6855
	p=.619	p=.177	p=.677	p=.621	p=.872	p=.760	p=.853	p=.679	p=.502	p=.217	p=.518	p= ---	p=.257	p=.096	p=.029
Hardness	-.0894	.6304	.6584	.1680	.1581	.0681	.2096	-.4347	-.2646	.2105	-.2400	.3964	1.0000	-.2576	-.0779
	p=.806	p=.051	p=.038	p=.643	p=.663	p=.852	p=.561	p=.209	p=.460	p=.559	p=.504	p=.257	p= ---	p=.472	p=.831
TVC	.1460	-.2425	-.5824	-.1741	-.1960	-.3791	.0989	.2481	-.2396	.6586	-.0247	-.5549	-.2576	1.0000	.8794
	p=.687	p=.500	p=.077	p=.631	p=.587	p=.280	p=.786	p=.489	p=.505	p=.038	p=.946	p=.096	p=.472	p= ---	p=.001
TYM	.0040	-.2841	-.3381	-.2726	-.2214	-.3781	-.0631	.1245	-.1901	.7345	-.1300	-.6855	-.0779	.8794	1.0000
	p=.991	p=.426	p=.339	p=.446	p=.539	p=.281	p=.863	p=.732	p=.599	p=.016	p=.720	p=.029	p=.831	p=.001	p= ---
